# Non-Convulsive Status Epilepticus and Mild Neurodevelopmental Phenotype in a Female with a Novel p.Thr657Ala Variant in the *GRIA3* Gene

**DOI:** 10.3390/children12121654

**Published:** 2025-12-05

**Authors:** Alfonso Rubino, Giorgia Bruno, Gabriella Errichiello, Fabio Acquaviva, Daniele De Brasi, Alfonsina Tirozzi, Pia Santangelo, Carmela Russo, Antonio Varone, Geremia Zito Marinosci, Pia Bernardo

**Affiliations:** 1 Pediatric Neurophysiology Unit, Department of Neurosciences, Santobono-Pausilipon Children’s Hospital, 80129 Naples, Italy; 2 Pediatric Neurology Unit, Department of Neurosciences, Santobono-Pausilipon Children’s Hospital, 80129 Naples, Italy; 3 Child Neuropsychiatry Unit, Department of Neurosciences, Santobono-Pausilipon Children’s Hospital, 80129 Naples, Italy; 4 Child Neuropsychiatry Unit, Department of Translational Medical Science, University of Naples Federico II, 80131 Naples, Italy; 5 Medical Genetics Unit, Department of General and Emergency Pediatrics, Santobono-Pausilipon Children’s Hospital, 80129 Naples, Italy; 6 Pediatric Neuroradiology Unit, Department of Neurosciences, Santobono-Pausilipon Children’s Hospital, 80129 Naples, Italy; 7 Pediatric ICU, Santobono Pediatic Hospital, 80129 Naples, Italy

**Keywords:** *GRIA3*, NCSE, X-linked epilepsy, neurodevelopmental disorders

## Abstract

**Highlights:**

*GRIA3* gene mutations seem to result in a broad spectrum of neurodevelopmental disorders, with heterogeneous epileptic manifestations, generally with early onset, potentially classified as the combination of a generalized type of epilepsy of genetic origin and focal seizures. Status epilepticus (SE) has been documented in previous reports, though only in male patients. This is the first reported case of NCSE in a female patient with a *GRIA3* variant. Overall, the clinical and genetic spectrum of *GRIA3*-related disorders remains poorly understood, especially in females. Our case shows the complexity of genotype–phenotype correlations. Further studies are needed to clarify variant-specific effects, sex-related expression differences, and potential therapeutic targets.

**What are the main findings?**

- Our case report showed a clinical history in a patient who, although with core symptoms consistent with the major features of the *GRIA3*-related disorder, presents a mild phenotype with a favorable epilepsy course.
- The observation of NCSE at onset remains peculiar, pointing out the potential role of the involvement of AMPA receptors in such critical manifestations.

**What is the implication of the main finding?**

- Our case shows the complexity of genotype–phenotype correlations.
- Further studies are needed to clarify variant-specific effects, sex-related expression differences, and potential therapeutic targets.

**Abstract:**

**Background:** The *GRIA3* gene encodes the GluA3 subunit of AMPA-type glutamate receptors, which are crucial for excitatory neurotransmission in the central nervous system. Pathogenic *GRIA3* variants cause X-linked neurodevelopmental disorders of varying severity, including developmental delay, behavioral abnormalities, and epilepsy. **Case Summary:** Here, we present the case of a seven-year-old female patient presenting with developmental delay, spastic gait, and non-convulsive status epilepticus (NCSE), who was found to carry a novel de novo *GRIA3* missense variant (c.1969A > G; p.Thr657Ala). The EEG revealed high-amplitude diffuse rhythmic theta/delta activity consistent with NCSE. A brain MRI showed transient cortical and thalamic T2-FLAIR hyperintensities, likely postictal. Metabolic investigations were unremarkable. Following intensive treatment with levetiracetam and midazolam, the patient gradually recovered to her baseline neurological status. **Genetic Finding:** Whole-exome sequencing (WES) identified a novel de novo variant in *GRIA3*, c.1969A > G; p.Thr657Ala, involving the replacement of threonine with alanine at position 657 within the coding region. **Significance:** This case expands the clinical and molecular spectrum of *GRIA3*-related disorders, demonstrating that females with de novo variants may experience severe epilepsy. This is the first reported case of NCSE in a female patient with a *GRIA3* variant.

## 1. Introduction

The *GRIA3* gene (Xq25) encodes the AMPA-type glutamate receptor (AMPAR) subunit 3, a member of the ionotropic glutamate receptor superfamily involved in excitatory neurotransmission. Assembly of GluA1-4 subunits into homo- or heterotetrametric AMPARs generates receptor subtypes with distinct biophysical properties across central nervous system (CNS) development [1,2,3].

Pathogenic *GRIA1*-4 variants can interfere with AMPAR physiology, leading to developmental delay, behavioral issues, seizures, and cerebral malformations. Whereas *GRIA1*, *GRIA2*, and *GRIA4* are autosomal genes in which pathogenic missense variants almost invariably arise de novo, *GRIA3* is located on the X-chromosome, and pathogenic variants may be transmitted from unaffected carrier mothers to affected male offspring, a pattern typical of several X-linked neurodevelopmental disorders (NDDs) [2,3,4].

Gain-of-function (GoF) variants in *GRIA3* are associated with severe phenotypes, including early-onset seizures, hypertonia, and hyperekplexia, while affected females most often carry de novo heterozygous GoF variants. However, the phenotypic spectrum of *GRIA3*-related disorders remains incompletely defined, especially in females [5,6].

Epilepsy has been reported in more than half of the cases, with heterogeneous ages of onset and clinical outcomes [1,3,4,6,7].

Here, we report a 7-year-old female with developmental delay, spastic gait, and non-convulsive status epilepticus (NCSE) carrying a novel de novo *GRIA3* variant. Nevertheless, clinical evolution appears favorable with good control of the seizures and mild overall functioning. Interestingly, this is the first reported case of NCSE in a female patient with a *GRIA3* variant.

## 2. Case Report

The proband, a female, was born small for her gestational age by cesarean section after an uneventful pregnancy. No perinatal distress or relevant family history was reported. At the age of two years, she exhibited developmental delay and lower limb spasticity. Paroxysmal episodes of unclear epileptic nature characterized by hypotonia, pallor, and behavioral arrest were also present.

No dysmorphic features were noted. Brain and spinal magnetic resonance imaging (MRI), electroencephalogram (EEG), and array-CGH were unremarkable. Over the following years, she developed a paraparetic spastic gait with hyperreflexia, expressive language impairment, and moderate intellectual disability (ID).

A cognitive evaluation using the WISC-V scale showed a score of 68 in “verbal comprehension”, 80 in “visual–spatial reasoning”, 46 in “working memory”, and 47 in “processing speed”. With regards to the Vineland Adaptive Behavior Scales, she showed a good profile in the “Communication” and “Socialization” areas, with worse capabilities in “Daily Living Skills”.

**Figure 1:**
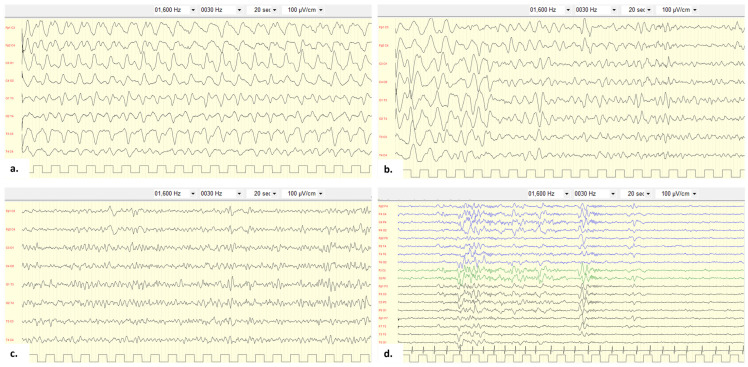
EEG of the proband. EEG performed in ICU revealing high-amplitude diffuse rhythmic shaped theta/delta activity (>10 s), predominant in the anterior areas (**a**), which improved after administration of midazolam in bolus (**b**,**c**). EEG performed before the discharge of the patient showing normal background activity without significant abnormalities (**d**).

At 7 years of age, she presented with atypical absences and drowsiness following azithromycin treatment for pharyngitis. On admission to the emergency department, she was comatose but responsive to pain (Glasgow Coma Scale 10/15), without focal signs. The cerebrospinal fluid (CSF) analysis was unremarkable, and infectious diseases were excluded. The EEG showed non-specific slow activity in the temporal area. During hospitalization, she developed bilateral tonic–clonic seizures, worsening hypertonia, and reduced consciousness, requiring transfer to the intensive care unit (ICU). She was treated with levetiracetam and midazolam, which resolved the clinical seizures. However, subsequent EEG recordings performed in the ICU showed electrical status epilepticus, characterized by high-amplitude diffuse rhythmic theta/delta activity without clinical manifestations, consistent with non-convulsive status epilepticus (NCSE) (Figure 1). The EEG fulfilled the Salzburg criteria for NCSE: continuous rhythmic theta–delta activity with fluctuating frequency and without definite evolution, which responded well to anti-seizure drugs [8]. Given the comatose state and EEG pattern, the patient was treated with midazolam, sodium thiopental, and propofol. NCSE lasted for 5 days, after which clinical conditions improved.

**Figure 2:**
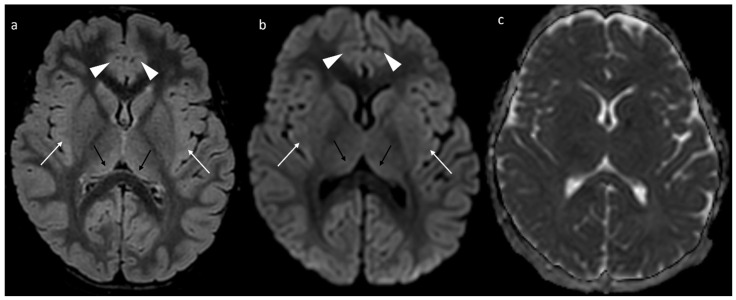
MRI study during status epilepticus shows FLAIR hyperintensity with mild swelling of the cerebral cortex (**a**), best seen in the insular and cingulate regions (white arrows and arrowheads), and the pulvinar regions of both thalami (black arrows). These regions demonstrate subtle diffusion abnormalities, with increased DWI signal (**b**) and without a significant drop in ADC values (**c**).

Brain MRI with contrast revealed T2-FLAIR hyperintensities involving the cortical profiles of both hemispheres, which were more pronounced in the insular region, anterior cingulate cortex, perirolandic area, and posterior thalami. These findings were considered secondary to seizure activity and were not associated with significant swelling (Figure 2).

Considering the severity of the clinical presentation, an extensive metabolic-wide screening was performed, including amino acids and neurotransmitters analysis on CSF. All results were within the normal range. Genomic DNA was extracted from peripheral blood using standard silica-column purification (Qiagen), following the manufacturer’s protocol. Coding regions and the exon–intron boundaries (±5 bp) of genes known to be associated with Mendelian disorders were analyzed. Gene selection was performed according to the clinical indication, using HPO, OMIM, and GeneReviews. Clinical exome sequencing (CES) was carried out in a trio setting (proband and parents) using the ClinEX Pro kit (4bases) for targeted enrichment, followed by paired-end sequencing on the Illumina NovaSeq 6000 platform. The assay provides an analytical sensitivity and specificity > 99%. The mean coverage of aligned regions was 267.51×, and only regions with a minimum depth of ≥30× were considered for variant interpretation. Raw reads were aligned to the human reference genome GRCh37 using the BWA Aligner/DRAGEN Germline Pipeline/DRAGEN Enrichment. Variant calling was performed through a standardized DRAGEN-based workflow. Variants were annotated and prioritized using Geneyx Analysis (GeneCards Suite), integrating allele frequency thresholds (gnomAD < 1%), predicted functional impact, inheritance model, and known disease associations (ClinVar, OMIM, HGMD). Only variants in the selected gene set and meeting established quality metrics were retained for analysis (according to Rehder et al., Genet Med 2021 [9]). Candidate variants were classified according to ACMG/AMP guidelines. When required, Sanger sequencing was performed to confirm the identified variants and to assess familial segregation.

**Figure 3:**
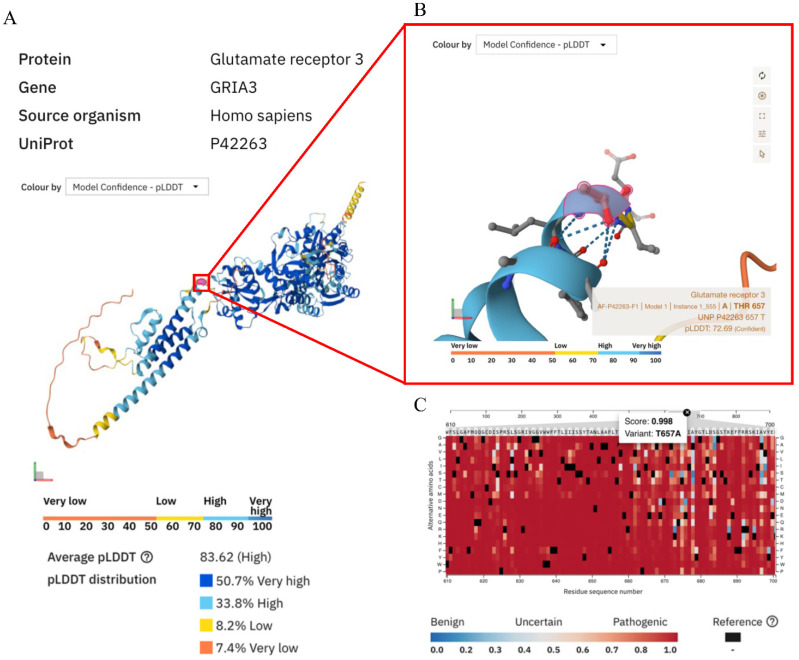
*GRIA3* 3D representation in alpha fold [10] (**A**) with zoom on amino acid (Thr657) conformation (**B**) and pathogenic score prediction in alpha fold (**C**).

Clinical exome sequencing (CES) identified a novel de novo variant in *GRIA3*, c.1969A > G; p.Thr657Ala, involving the replacement of threonine with alanine at position 657 within the coding region. This is a missense variant absent from the gnomAD database (allele frequency = 0%). The pathogenicity of the variant is supported by silico prediction tools, which suggest a deleterious effect. According to American College of Medical Genetics and Genomics (ACMG) guidelines, it is currently classified as likely pathogenic based on PM1, PM2, and PP3 criteria. We performed a pathogenicity prediction using the AlphaFold protein structure database (https://alphafold.ebi.ac.uk, accessed on 5 October 2025) (Figure 3), which confirmed the deleterious effect of the variant with an average AlphaMissense pathogenicity score of 0.998. This score represents the mean predicted pathogenicity of missense variants within a given protein region. Scores range from 0 (benign) to 1 (pathogenic) and are calculated using the AlphaMissense model, which uses AlphaFold predictions to derive structural, evolutionary, and biochemical features.

Recovery was slow, taking approximately 4 weeks, but the patient returned to her baseline status, characterized by a mild paretic gait, oral feeding, and a normalized EEG at discharge.

## 3. Discussion

*GRIA3* gene mutations seem to result in a broad spectrum of neurodevelopmental disorders with heterogeneous epileptic manifestations [1,3,4,11]. To date, around 50 patients with *GRIA3*-related disorders have been reported, including about 22 females [1,2,11,12].

The neurological phenotype is generally severe, with a combination of hypotonia, hypertonia, movement disorders, and hyporeflexia [1,2,3]. Other findings may include cerebellar involvement (ataxia, tremor, and dysmetria) [3,4,5], macrocephaly, sleep disorders, hyperekplexia, and erratic nonepileptic myoclonus [4,6,7,13,14]. Dysmorphic features have also been reported, including horizontal eyebrows, epicanthic folds, a short nose, prominent nasolabial folds, and a tented upper lip [15,16,17]. Additional traits may include brachycephaly, deep-set eyes, prominent supraorbital ridges, foot anomalies, malar flattening, hypotonic facies, prognathism, and oral anomalies such as a narrow palate or prominent incisors. MRI findings are similarly variable, ranging from normal to several non-specific anomalies such as cerebellar vermis hypoplasia, right frontal cortical dysplasia [3,4,5,6], frontal atrophy, ventricular enlargement, a thin corpus callosum, and retrocerebellar cysts.

The wide variety of symptoms and severity, remarkable in the GRIA-3 related disorder per se, tend to also be more evident in female subjects, characteristically partially ascribable to X-linked recessive inheritance [6].

Epilepsy occurs in about 40% of patients with a generally early onset (<3 years), although exact ages are not always specified. Heterogeneous seizure manifestations are reported, although phenotypical electro-clinical descriptions are not always adequately characterized. However, *GRIA3*-related epilepsy seems potentially classified as the combination of generalized and focal seizures with genetic origin.

EEG characteristics typically include disorganized background activity [1,4,5,7], bilateral high-voltage discharges, and epileptiform abnormalities predominating in the frontal or central regions, often progressing to high-amplitude diffuse spike-and-wave patterns [3,4,5].

Status epilepticus (SE) has been documented in two cases. Trivisano et al. described a male patient with ID, non-convulsive SE, myoclonia, and spasms, who proved resistant to multiple therapies [5]. Rinaldi et al. reported an 11-year-old boy with ID, recurrent myoclonic SE, cerebellar syndrome, vermis hypoplasia, and fronto-central dysplasia, who responded well to ethosuximide and clobazam from the age of 14, despite persistent EEG abnormalities [4]. Notably, there are two reports of SE involving different pathogenic variants affecting the same amino acid residue. Additionally, this is the first reported case of NCSE in a female patient with a *GRIA3* variant.

The previous reports present substantial differences in epileptic severity and neuroimaging findings compared to our case [16,17,18,19]. Indeed, the patient, although with core symptoms consistent with the major features of the malady, presents a mild phenotype with a favorable epilepsy course. In these terms, the observation of a NCSE at onset remains peculiar, pointing out the potential role of the involvement of AMPA receptors in such critical manifestations. It is worth considering that the trial of perampanel, an antiepileptic drug with the AMPA receptor mechanism of action, did not prove clinical or EEG improvement in a previous report [1,3,4,5]. However, the pathway from a molecular diagnosis to the administration of a pathophysiology-based treatment is only partially applicable in epilepsy, considering the complex interactions at the various biological levels that these conditions may generate [20]. Nevertheless, detailed outcome data and treatment responses at the moment are scarce [5,19,20].

The pathogenic mechanism of our novel p.Thr657Ala missense variant remains to be established. While it is not possible to determine whether the variant exerts a gain- or loss-of-function effect, a gain-of-function mechanism appears more likely, taking into account that female patients more frequently harbor such variants [4,5,6,7,8].

However, much more devastating phenotypes are reported in the literature than in our patient’s presentation. In fact, some authors speculated that mixed *GRIA3* variants may lead to a more severe phenotype compared with what has been associated with GoF effects only.

**Figure 4:**
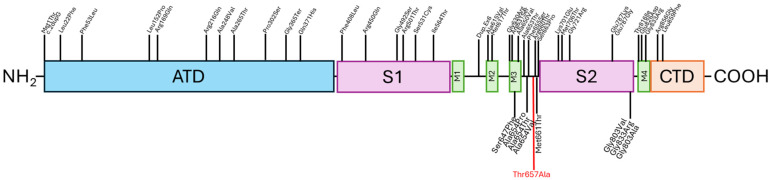
*GRIA3* schematic domains representation showing previously reported pathogenic variants (in black) and the novel variant identified in this study (in red). The diagram illustrates the spatial relationship and potential impact of the variants.

To contextualize the novel p.His657Ala variant, we compared the clinical phenotype of our cases with published *GRIA3* hotspot variants (Figure 4), notably p.Gly787 and p.Gly803. Reported cases carrying p.Gly787 and p.Gly803 typically present with early-onset epileptic encephalopathy, severe developmental delay/intellectual disability, and prominent motor impairment; electrophysiological and functional studies of these hotspots suggest altered AMPA receptor gating consistent with gain-of-function (GoF) effects [4]. By contrast, carriers of p.His657Ala in our cohort display a milder neurodevelopmental profile characterized by later seizure onset, attenuated cognitive impairment, and retained (albeit delayed) motor milestones. Structurally, p.His657Ala maps to the linker region between the ligand-binding domain and the transmembrane domain—an area implicated in LBD–TMD coupling—whereas p.Gly787 and p.Gly803 lie closer to pore-forming or gating elements; this spatial separation supports the hypothesis that p.His657Ala may perturb receptor function through a distinct (and potentially less disruptive) mechanism. We therefore propose that p.His657Ala could exert a subtler modulatory effect on receptor kinetics than classic hotspot GoF variants, although definitive classification requires electrophysiological assays and expression studies.

The comparatively mild clinical presentation observed in the female carriers of the p.His657Ala variant is consistent with the known sex-related modulation of *GRIA3*-associated phenotypes. As an X-linked gene, *GRIA3* is subject to X-chromosome inactivation (XCI), which generates mosaic expression in females and results in a mixed neuronal population expressing either the wild-type or the mutant allele. This mosaicism is a well-established mechanism underlying phenotypic attenuation in female carriers of X-linked neurodevelopmental disorders [8]. The clinical data from our patients, including later onset of seizures, milder neurodevelopmental impairment, and partial preservation of motor and cognitive milestones compared with male cases reported for hotspot variants such as p.Gly787 and p.Gly803, are compatible with the buffering effect of XCI. Furthermore, *GRIA3* exhibits sex-biased expression patterns in human brain tissues, particularly during early neurodevelopment, which may further modulate variant penetrance and severity. In this context, even subtle differences in allelic expression ratios could mitigate the functional consequences of variants with moderate structural impact, such as p.His657Ala, which lies outside the canonical hotspot regions associated with severe gain-of-function mechanisms. Although XCI testing was not performed at the time of initial evaluation, we acknowledge its relevance and plan to incorporate it in follow-up analyses, alongside functional studies, to better quantify the contribution of X-linked mosaicism to the observed female phenotype [10]. Overall, the clinical and genetic spectrum of *GRIA3*-related disorders remains poorly understood, particularly in females, where phenotypic variability is substantial and often counterintuitive given the X-linked inheritance pattern (see Table 1). Our case highlights the complexity of genotype–phenotype correlations, illustrating a comparatively mild neurological and epileptic presentation despite harboring a novel missense variant. This observation not only expands the phenotypic spectrum associated with *GRIA3* variants but also underscores the critical need for detailed clinical and molecular characterization to understand sex-specific effects, variable expressivity, and potential mechanisms modulating the phenotype in female carriers.

## Figures and Tables

**Table 1:** Phenotype–genotype features and a literature review.

Author/Year	N/Sex	Age	E	E Onset (mo)	E Semeiology/DR	ASM	Np	MRI	Inheritance	Genotype
**Fons et al., 2025 [11]**	1/F	1 mo	+	1	FS TS/Yes	PHB, VPA, LEV, OXC, CZP, LCM, KD	DD, microcephaly, parkinsonism.	Polymicrogyria	De novo	c.1951A > G
**Sjøstrøm et al., 2025 [1]**	6/F (4)	12 Y 33 Y 10 Y (2) 3 Y 5 Y	+ (2 M + 1 F)	1 (M); 11 (M); 6 (1 F)	FBTCS (M); My (1 M + 1 F)	NA	ID (6); Cerebral palsy (4); Startle (6); Hypertonia (6); DEE (1 M); EIMFS (1 M)	Abnormal (1 F)	De novo (5); Mother (1)	c.2408G > A c.2408G > T c.2408G > C
**Rinaldi B et al., 2024 [4]**	25/F (11)	-	+ (12)	1–16	FS (7) Un (4) Ab (2) My (5) GTC (1) A (1)	NA	ID; LMHy (13); H (10); MD (14); DF	Abnormal (4)	De novo	c.159T > G c.506G > A c.743C > T c.904C > T c.941T > C c.1093G > T c.1113A > T c.1224C > G c.1349G > A c.1474G > A c.1502G > C c.1592C > G c.1691T > C c.1844C > T c.1850T > C c.1888G > C c.1891C > A c.1940C > T c.1957G > A c.1960G > C c.1960G > A c.1961C > T c.1987T > C c.2101A > G c.2408G > A c.2408G > C c.2447C > T c.2477G > A c.2497G > C c.2566C > G c.2575C > T
**Okano S et al., 2023 [7]**	1/F	13Y	+	3	FS CS TS/Yes	CBZ, LTG, CLB, LEV, LAC	ID; H	Mild frontal lobe atrophy and slight ventricular enlargement	De novo	c.1982T > C
**Necpál J et al., 2023 [12]**	1/F	-	+	NA	-	NA	ID; MD	-	De novo	c.1949C > T
**Martinez-Esteve Melnikova A et al., 2022 [2]**	1/F	-	+	NA	-	NA	-	-	De novo	c.2359G > A
**Rinaldi B et al., 2024 [4]**	1	11 Y	+	29	MySE My CS/Yes	VPA, ETS, CLB, ACTH, Hy, IGIV, Bromide, CLN	ID; Cerebellar syndrome: ataxia, tremor and dysmetria, DF	Cerebellar vermis hypoplasia and cortical dysplasia in right frontal region	Mother	c.2360A > G
**Sun JH, 2021 [3]**	1/F	1Y	+	NA	-	NA	ID	-	De novo	c.1979G > C;
**Epilepsy Phenome/Genome Project, Epi4K Consortium, 2021 [13]**	1	6Y	+	NA	-	NA	ID	BPP	-	c.2359G > A
**Piard J et al., 2020 [21]**	4	15 (3); 24 (1)	No	no	no	no	Mild ID, generalized chorea, multifocal myoclonus	Normal	Mother	c.2477G > A
**Trivisano et al., 2020 [5]**	1	2Y2mo	+	16	Ab, My, NCSE/Yes	ACTH. ICU: Mi, Ke, SoTh, Pro. TPM, PER	ID; Asthenic body habitus, poor muscle bulk, distal Muscle weakness, hyporeflexia; DF	Normal	Mother	c.1957G > A
**Davies et al., 2017 [14]**	2	27Y; 30Y	-	NA	-	-	ID; SD	NA	Mother	c.1964T > C
**Chérot et al., 2017 [15]**	1	-	NA	NA	-	-	ID	NA	Mother	c.743C > T
**Allen et al., 2016 [16]**	1/F	7Y	+	6W	FS, CS, TS, IS	-	ID	Normal	De novo	c.1888G > C
**Philips et al., 2014 [22]**	3	57Y; 35Y; 36Y	+	NA	-	-	ID; Hy, pain insensibility, scoliosis, testicular ectopia, DF	NA	Mother (7)	dup 1.2-Mb
**Philips et al., 2013 [22]**	2	24Y; 4Y6mo	+	NA	-		ID; Hy, joint laxity, valgus pes planus, genu recurvatum, micropenis, DF	Thin CC, atrophy of the last 2 lobules of the cerebellar vermis	Mother	dup 5.02-Mb
**Bonnet et al., 2009 [17]**	2	20Y	-	NA	-	ID; DF	Retrocerebellar cyst (1)	Retrocerebellar cyst (1)	Mother	dup 0.275-kb
**Guilmatre et al., 2009 [23]**	1	-	-	NA	-	ID; ASD	NA	NA	Mother	dup 1.42-Mb
**Chiyonobu et al., 2007 [18]**	1	4Y7mo	-	NA	-	ID; ASD; DF	NA	NA	Mother	dup 0.498-kb
**Wu et al., 2007 [19]**	5	-	+ (1)	NA	My	ID; ASD; poor muscle bulk, hyporeflexia (1); macrocephaly (1); asthenic body habitus (2), poor muscle bulk, distal muscle weakness, hyporeflexia	NA	NA	Mother	del 0.4-Mb c.2497G > C c.2117T > C c.1891C > A c.1349G > A
**Gécz et al., 1999 [24]**	1	20Y	+	18	GTC	ID	NA	NA	De novo	t(X;12) (q24; q15)

Ab: absence; A: atonic; ASD: autism spectrum disorder; BPP: bilateral perisylvian polymicrogyria; CS: clonic seizure; DD: developmental delay; DF: dysmorphic features; DR: drug resistance; E: epilepsy; FS: focal seizure; GTC: generalized tonic–clonic; H: hypertonia; IS: infantile spasm; ICU: intensive care unit; ID: intellectual disability; Ke: ketamine; LMHy: limb muscular hypotonia; M: moderate; Mi: midazolam; My: myoclonic; MD: movement disorder; N: number; NA: not available; Np: neurological phenotype; SD: sleep disorder; SoTh: sodium thiopental; TS: tonic seizure. the recent manuscript by Rinaldi et al. includes seven previously published patients on a series of 25 subjects. (Rinaldi B et al., 2024 [4]; Sun JH, 2021 [3]; Trivisano et al., 2020 [5]; Davies et al., 2017 [14]).

## Data Availability

No datasets were generated or analyzed for this case report. All information presented in the manuscript is contained within the article.
